# Analysing the Contributions and Longevity of Community Archaeology in the Context of Maritime Cultural Heritage Projects

**DOI:** 10.1007/s11457-021-09318-9

**Published:** 2021-12-20

**Authors:** Makanani Bell, Lucy Blue

**Affiliations:** Centre for Maritime Archaeology, University of Southampton, Southampton, UK

**Keywords:** Community archaeology, Evaluation, Maritime heritage, Heritage management

## Abstract

Scholars readily agree community archaeology offers a way to engage non-professional archaeologists in the archaeological process. However, few analyse whether community archaeology projects achieve their goals and contribute positively to involved stakeholders. This article proposes a framework for analysing the contributions and longevity of community archaeology in maritime environments. The framework consists of three sections: the influencing factors, intended and actual contributions, and longevity. The influencing factors highlight the most common elements that impact the contributions of a project. The intended and actual contributions compare the project’s goals with their results. The longevity section proposes a number of prompting questions to assess the longevity of the outcomes. Three case studies provide a closer look at each project’s influencing factors, contributions and longevity. Synthesizing the case study’s results reveals several overall conclusions and areas for improvement within community archaeology.

## Introduction

Theoretical advances in the last thirty years have broadened the field of maritime archaeology to include the cultural landscapes of lacustrine, riverine, and maritime environments (Westerdahl 1992: 5; Ford et al. 2013: XIV; Meide 2013: 12). These advances encourage incorporating physical and cognitive landscapes and the inclusive documentation of all peoples, heritages, and knowledge sources. In turn, this values and records both tangible and intangible heritage. Intangible heritage is often passed from one generation to the next (UNESCO 2011). Acknowledging the importance of intangible heritage underpins the inclusion of community in the archaeological process through acknowledging community members as resources and valuing community-held knowledge, such as oral histories, place names, traditions of usage, and legends (Chirikure and Pwiti 2008: 474; Freire 2014: 114). The information gained tells a more complete story of the past through incorporating details frequently missed, such as the elements that instil in people their sense of place and identity (Little 2009: 117; Greer 2010: 53; Nero 2011: 12; UNESCO 2011).

Many different approaches to community archaeology exist with a range of interpretations and definitions. The most widely used definition of community archaeology is where the archaeological process engages non-archaeologists in some capacity (Belford 2014: 23). Community archaeology projects take different forms, adopt a variety of different methodological approaches and engage with a wide range of communities. However, they share similar strategies and goals. Each one seeks to counter the colonial paradigm, tell more inclusive stories of the past, and demonstrate that archaeology can achieve more than collecting artefacts (Kusimba 2017: 218; Wright and Kod 2011: 115). Recognizing the community as partners in heritage investigations addresses frequently overlooked power imbalances present in archaeology and restores agency to the communities associated with the heritage in question (Roberts et al. 2013: 78; Supernant and Warrick 2014: 566; Guilfoyle and Hogg 2015: 6). This wide definition of community archaeology overlaps with other types of archaeological research that engages non-archaeologists such as public archaeology, collaborative archaeology, and participatory action research. The distinction between these types of research is not always definitive but each project assessed in this paper self-identifies as community archaeology and is thereby included.

Scholars have long promoted the benefits of community archaeology (e.g. Marshall 2002; Moser et al. 2002; Chirikure and Pwiti 2008; Little 2009; Atalay 2012). Some of these benefits include enhancing community pride, identity, and unity in their heritage and each other (Wright and Kod 2011: 115; Roberts 2016: 228). Community archaeology also helps build cultural stewards, nurture public support for heritage, and ensure the longevity of archaeology as a whole (Belford 2014: 40; Fletcher 2014: 5; Chinyele and Lwoga 2019: 184). Despite the benefits attributed to this method, few archaeologists have evaluated the effects and sustainability of their work, determined whether the project is successful for all stakeholders, and established if anyone has been negatively affected. Fewer still have made this research publicly accessible. Archaeologists must begin evaluating the contributions and if possible, the longevity of their work, in order to understand its impact on all involved stakeholders and the duration of these affects.

This paper proposes a methodology to analyse community archaeology projects. Extensive background reading, as referenced, and dialogue with community archaeology practitioners, informed the creation of this framework. The framework consists of three sections: the influencing factors, intended and actual contributions, and longevity. The influencing factors highlight ten of the most common elements of community archaeology that impact the contributions a project can make. The second section compares intended and actual contributions to all stakeholders. The third section provides a set of questions that prompt a discussion about the longevity of the project’s outcomes. The framework was tested on five case studies as a part of a larger study, three of which are presented here. This analysis revealed conclusions about the framework and community archaeology itself.

The authors are keenly interested in maritime archaeology and previously no evaluation framework for community maritime archaeology existed. This research thus focused on providing a solution. However, the framework presented in this paper could easily be applied to terrestrial archaeology and the heritage management sector. The primary aim is to promote a more inclusive approach to community maritime archaeology and begin a much-needed conversation regarding its evaluation and longevity. However, this framework, as a derivative of the development of archaeological practice more generally, also leaves areas for improvement. It is fundamentally driven by archaeologists, and by a need to improve academic evaluation, and as such runs the risk of extending a hierarchical approach and non-inclusivity of stakeholders, at least in the project conception and design. Thus, the intention is that by raising the profile of a more integrated approach, hierarchies will be eroded and a truly bottom up, comprehensively evaluated process can emerge, that engages, where possible, the full range of stakeholders for the benefit of the entire community and archaeological resource alike.

Although we set out to create an evaluation tool, the resulting framework can and should be used in both designing and evaluating community maritime archaeology by any stakeholder in any project. Using this framework in designing projects will help users create more inclusive, successful, and impactful projects. Using the framework in evaluating projects will help users understand the intended and unintended consequences of their research and the longevity of the impact. Thus, the framework can used to design and evaluate any project.

## Theoretical Framework

Westerdahl’s (1992) theory of maritime cultural landscapes brought about significant changes to maritime archaeology. This theory helped break down arbitrary divides between land and sea, acknowledging how people and their ideas flow freely between these spaces, and encouraging maritime archaeologists to investigate both sides of the waterline (Cobb and Ransley 2019: 19; McKinnon et al. 2014: 61). Through recognizing the physical and cognitive landscape, maritime archaeology became more inclusive; widening the areas studied, people and stories included, and knowledge sources utilized, including valuing both tangible and intangible heritage (Westerdahl 1992: 5; Ingold 1993: 172; Carter 2011: 9; Ford 2011: 1; McKinnon et al. 2014: 61). In turn, these advances encouraged the involvement of the public and communities in the archaeological process through community engagement, following the development of community archaeology in terrestrial studies.

Theories of landscapes and seascapes support inclusivity in two ways. Firstly, these theories support including a diverse range of people in archaeological studies, from occupations not originally discussed in maritime studies to societies omitted from accounts of the past for discriminatory reasons. Secondly, inclusivity extends into the kinds of knowledge sources considered and accepted as valid. Traditional approaches to scholarship focus on building knowledge based upon previous academic outputs. This approach often overlooks knowledge generated through sources including local people, elders, oral histories, and ethnographies (Chirikure and Pwiti 2008: 470). Inclusively studying and valuing these resources helps tell a more complete account of the past, encourages interdisciplinary studies, and documents stories, peoples or heritages not traditionally discussed within maritime archaeology or excluded for discriminatory reasons (Westerdahl 1992: 5; Chirikure and Pwiti 2008: 474; Ford 2011: 1; McKinnon et al. 2014: 61). Widening the resources consulted encourages studying and valuing intangible heritage alongside the more traditionally considered tangible heritage.

Archaeologists are essentially focused on the documentation of tangible heritage. In contrast, intangible heritage (i.e. place names, legends, songs) is often overlooked (Liston et al. 2005: 184; Freire 2014: 144). Theories of maritime cultural landscapes acknowledge the intangible heritage associated with the physical site (Westerdahl 1992: 5; Ford et al. 2013: XIV; Meide 2013: 12; Sharfman 2017: 83). Alongside this development came a shift from viewing heritage as a monument, a physical object with historic and aesthetic qualities, to including the intangible landscapes within and surrounding the site (Lwoga 2018: 1019). Inclusively researching tangible and intangible heritage tells a more complete story of the past, including elements such as those that instil in people their sense of place and identity (UNESCO no date; Little 2009: 117; Greer 2010: 53; Nero 2011: 132). People often pass intangible heritage from one generation to the next, generating knowledge that becomes ‘traditional, contemporary, and living at the same time’ (UNESCO no date). These living, intangible heritages are held within the hearts and minds of present communities.

Additionally, although people occupy the same physical spaces over generations, the cognitive landscape alters between individuals and communities over time. A person may live in the same physical site as their ancestors, but they view and associate with the space differently (Ingold 1993: 162). This indicates how heritage is constantly living and changing through time. This concept encourages the incorporation of community into research and management strategies as their knowledge offers a primary source of intangible heritage. Archaeologists have turned to community engagement to access, understand, and incorporate intangible heritage through generations. Community engagement can deepen the kinds of research questions asked and answers received, while simultaneously benefiting all stakeholders (Nero 2011: 130; Wright and Kod 2011: 115; Kusimba 2017: 220).

People engage with the past to help establish meaning in the present (Marshall 2002: 211). Archaeologists produce knowledge associated with the past and thereby hold significant voice and power in the present. Power over the representation of the past is the responsibility of archaeologists who often overlook the community as a resource of knowledge with respect to the archaeological sites, often only incorporating these people when they are in need of labour. Directly involving the community in the research process changes the community from passive agents, an afterthought in the archaeological process, to active agents, crucial for the success and mission of the project (Marshall et al. 2009: 233). This restores voice to the community and recognizes them as an authority on their own heritage (Chirikure and Pwiti 2008: 472). Depending on the nature of engagement, this can potentially shift the balance of power from the archaeologists to the community members themselves, restoring their agency (Roberts et al. 2013: 78; Supernant and Warrick 2014: 566; Guilfoyle and Hogg 2015: 6). Community involvement also holds the archaeologists more ethically accountable than more traditional methodologies (McKinnon et al. 2014: 64).

Archaeology has the power and potential to impact communities today positively and negatively. Involving community in the archaeological process amplifies this potential and can both stimulate positive change but also can cause significant harm to involved stakeholders (Supernant and Warrick 2014: 584). Engagement can help instil the skills and passion for people to become stewards of heritage, recognize community members as an authority on their heritage or in their local area, enhance community pride and cohesion, stimulate generational knowledge transmission and more (Chirikure and Pwiti 2008: 472; Wright and Kod 2011: 115; Fletcher 2014: 5; Roberts 2016: 228). Engagement can also fuel feuds between neighbouring communities by setting apart community members by including some and excluding others, trigger land and resource ownership disputes, over-tax communities, build reliance on project money, and more (Supernant and Warrick 2014: 580; Woodfill and Rivas 2020: 572). Furthermore the effects of community archaeology may not finish at the end of the project, rather they can last well beyond a projects’ completion (Ellenberger and Richardson 2018: 79).

Thus, through community engagement, sharing accounts of projects, and understanding and reporting on positives and negatives of projects, greater benefit will ensue for the project and the community alike. Conducting evaluations will aid in this endeavour.

## Evaluation

### Why do We Need to Evaluate?

Evaluating community archaeology projects would help determine if the work is effective in determining its goals for all stakeholders including the archaeologists and community at large. Evaluations can identify if the research achieves the stated outcomes and analyse the effects of the research long term (Gould 2016: 8; Ellenberger and Richardson 2018: 67). Assessments would highlight people who are positively and negatively, impacted from the engagement activities. Furthermore, the evaluations would help provide an understanding of which methods achieve the desired or undesired outcomes (Gould 2016: 8). This information would highlight successes and areas for improvement, helping archaeologists recognize, understand, and report their project’s consequences (Fredheim 2018: 622). In turn, evaluations could help advance theories, and methods in relation to successful community archaeological practice for practitioners and communities alike. This would progress the discipline and help future projects chose methods that benefit communities and the acquisition and interpretations of archaeological data (Guilfoyle and Hogg 2015: 4; Ichumbaki 2015: 530). Furthermore, evaluations would also help archaeologists demonstrate the impact, credibility, and value of their work across the range of stakeholders (Kajda et al. 2017: 1; Ellenberger and Richardson 2018: 82).

### How do We Evaluate?

To date, little published work states exactly how scholars, as well as practitioners, community members and managers, should evaluate community archaeology in terrestrial environments (Guilfoyle and Hogg 2015; Heritage Lottery Fund 2019; Simpson and Williams 2008; Simpson 2009, 2008; Lewis 2014), with even less published about community engagement in maritime landscapes. Several characteristics of community archaeology contribute to the challenges of evaluation as outlined in the following paragraphs. Firstly, community archaeology lacks a uniform methodology. Secondly, articles frequently omit the methods used. Thirdly, results can be both tangible and intangible. Finally, the contributions themselves pose a challenge to measure as results are indicated in qualitative and quantitative fashions.

A singular, uniform methodology for community archaeology does not exist as each project has different needs and requirements. Instead, scholars have outlined ‘hallmarks’ of successful or meaningful community archaeology projects (e.g. Moser et al. 2002; Nicholas 2008; Atalay 2012). These are widely recognized as fundamental protocols of working alongside communities. Adaptability encourages archaeologists to tailor their methods to fit the project needs. However methods thereby differ, making evaluating a project against a framework of ‘successful’ collaboration difficult (Guilfoyle and Hogg 2015: 4).

Within published community archaeology projects, a lack of reporting or sharing the methodology employed, compounds this issue. Publications generally take a case study format and usually omit thorough discussion of the methodology used and a reflection on the efficacy of the study (Guilfoyle and Hogg 2015: 2; Gould 2016: 7; Ellenberger and Richardson 2018: 70). Literature reviews therefore fail to encompass the wide range of community research conducted and readers only receive anecdotal accounts of the projects’ results (Gould 2016: 11; Ellenberger and Richardson 2018: 70).

Gould examined 191 articles published in Volume 1(1) in 2000 to Volume 14(1) in 2015 in *Public Archaeology*. Ninety-seven papers presented an extensive discussion of one or multiple case studies (Gould 2016: 111). Only twenty-two of these papers described their methodology in any form. Without an understanding of the method used, it is difficult to understand how the various outputs and conclusions were achieved. These components increase the readers’ level of understanding and potential learning from the project, as well as provide critical information for meta-analysis.

Gould’s investigation also found, with a few exceptions, that research results presented in articles are largely given in an impressionistic form without detailed supporting data or anchors in theoretical models or hypothesized outcomes (Gould 2016: 111). Furthermore, rarely do these papers critically examine the case studies reported on. Only two papers (Simpson and Williams 2008; Lewis 2014) of the 191 offer evaluations (Gould 2016: 11). Although this research only provides an analysis of one academic journal, it gives a glimpse into the information available to community archaeology practitioners. Readers would gain more if authors described their methods, provided evidence to support their conclusions and outcomes, and evaluated their work. This information would help archaeologists learn from one another and simultaneously advance the field (Gould 2016: 14).

The mechanics of conducting an evaluation is further challenged as a project’s outcomes can be either tangible or intangible. For example, a community archaeology project that conducts archaeological surveys and provides an avenue for dialogue between community members and archaeologists could produce tangible site plans and improve understandings of the physical heritage. The project could also contribute intangibly through decolonizing history and fostering the preservation of community knowledge through encouraging transgenerational knowledge transfers.

Lastly, the dynamic and varied potential contributions cannot all be neatly measured quantitatively or qualitatively. Quantitative data collection involves producing numerical results and provides a compact way to represent abstract ideas, frequently measuring the quantity of something rather than its quality (Neuman 2014: 204; Stevenson and Lindberg 2017). Quantitative studies often involve closed questioning methods, with predetermined questions (Simpson 2009: 116). This method of inquiry can, unintentionally or intentionally, insert biases and guide respondents towards particular answers, implicating the validity of the study (Simpson 2009: 117). Additionally, questionnaires can run the risk of listing learning outcomes and asking whether the participants thought they were achieved, rather than assessing how they felt or their experiences over all (Simpson 2009: 118). Quantitative studies thereby can omit assessing the more personal, intangible aspects of heritage.

Qualitative data collection often includes gathering spoken or written words, actions, sounds, symbols, visuals, and more. The process leaves information in a non-standardized, diverse format, rather than converting the results into a single medium, as with quantitative data collection (Neuman 2014: 204). Qualitative data collection has the opportunity to explore the intangible contributions of heritage, such as the social or cultural benefits. Additionally, it emphasizes the complexity of a situation and encourages personal, individual responses from participants (Simpson 2009: 119). Qualitative studies can be more time consuming and are often difficult to statistically analyse. A hybrid of qualitative and quantitative methods might prove most effective in evaluations.

Publications by the Heritage Lottery Fund (HLF) (Heritage Lottery Fund 2019), Simpson (2008, 2009; Simpson and Williams 2008), Lewis (2014), and Guilfoyle and Hogg (2015) provide some of the only examples of evaluation methods for community archaeology. Each of these methods and others were evaluated, revealing successes and shortcomings. Understanding these and reading widely, helped the authors understand what scholars want evaluations to include.

The HLF requires evaluation through their grantees’ entire project, starting with gathering baseline data at the beginning (Heritage Lottery Fund 2019). HLF sets out rough guidelines and recommendations for analysing the project’s outcomes to heritage, people, and communities. Grantees are encouraged to use a variety of qualitative and quantitative measurements to understand the impacts of their work (Heritage Lottery Fund 2019). In turn, these evaluations help demonstrate the success of HLF’s financial contributions in their organization’s self-evaluations. Despite the fact that HLF is one of the few funding bodies that demonstrates good practice and requires monitoring and evaluation, it could still benefit from improvement, such as integration of standardized frameworks and a comprehensive synthesis of previous evaluations (Gould 2016: 12; Ellenberger and Richardson 2018: 75).

Simpson’s (2009) evaluation focused on assessing the espoused and actual values of several case studies, using both self-reflexive analysis and ethno-archaeology (Simpson 2008: 4; Simpson and Williams 2008: 71). This evaluation method therefore works well when employed from the beginning of the project through to completion. However, it is not so effective when used retroactively because a successful approach relies on ethnographic methods and conversations with participants to be undertaken during the project, which would not be possible to conduct with the same kinds of results after the project finished.

Lewis (2014) presents evaluations of the Higher Education Field Academy (HEFA), a longer-term community archaeology project. Evaluation begins with potential participants’ initial application to the program and continues throughout the project’s duration, gathering qualitative and quantitative data. Follow up evaluations are conducted several years after the students’ participation (Lewis 2014: 301). The evaluations help assess the project’s engagement, its long-term impacts and identifies areas where change could be encouraged (Lewis 2014: 310). This method may only work in community engagement projects that gather applications, written assignments, and keep in contact with participants in the long term.

Hogg’s (Guilfoyle and Hogg 2015) evaluation method tests five attributes of community archaeology projects on a scale of high, medium, low, or not present. This method analyses the level of engagement and community support the project had, as well as indicates whether the needs of the involved parties were met (Guilfoyle and Hogg 2015: 11). This method does not take into consideration the opinions of all collaborators.

Guilfoyle presented a methodology that quantitatively analyses the qualitative aspects of collaborative cultural heritage management (Guilfoyle and Hogg 2015: 21). This method analysed projects individually, while providing a framework to compare and evaluate projects against one another (Guilfoyle and Hogg 2015: 23).

The aforementioned frameworks provide different ways to collect information, analyse projects, and present results. Studying these evaluation models and others, influenced the development of the framework presented in “The methodology” section, and informed our understanding of what evaluations needs.

### What Does an Evaluation Need?

As outlined above, several potential evaluation methodologies exist including employing ethnography, studying single case studies, conducting a meta-analysis, or undertaking a large-scale case-study investigation. An evaluation of the extant data cited here and listed in the bibliography, revealed that community-based archaeological projects and hence any subsequent evaluation of such projects, should include six key elements:

1. Identify for whom the project is being conducted and why,
2. Include all stakeholders’ voices,
3. Clearly identify the level and duration of engagement,
4. Report on successes and failures,
5. Seek to understand the methodology behind achieving each outcome,
6. And evaluate in an unbiased fashion.

1. *Identify for whom the project is being conducted and why,*

What denotes ‘success’ varies between projects. All involved parties should clearly identify what ‘success’ means to their project together, identify for whom the project is being conducted, and why (Atalay 2012: 254; Fredheim 2018: 626). Conversations with stakeholders before, during, and after the project will deepen the understanding of the social, economic, and educational needs of all involved (Ellenberger and Richardson 2018: 82). This important information directly impacts the project’s outcomes and the relationships of those involved during the project and into the future.

2. *Include all stakeholders’ voices*

Just as scholars call for an inclusive archaeological approach incorporating numerous voices (Kajda et al*.* 2017: 20), evaluations should consciously include the voices of community members, project leaders, and all stakeholders in the entire evaluation process (Atalay 2012: 80). Not considering each stakeholder potentially inserts biases and could miss essential consequences of the project. Inclusion of all stakeholders starts at the very inception of the project, preferably through a process of co-creating a project design that recognises the needs of all partners and negotiates how to prioritize and achieve the greatest benefit for all where possible.

3. *Clearly identify the level and duration of engagement*

Evaluations should take into consideration who the ‘community’ is, how they are engaged, and the duration of engagement (Cornwall 2008: 280). Levels of engagement differ significantly between different kinds of community archaeology and with different sections of the community. Evaluations should clearly state the level and duration of engagement and which members of the community participated and how engagement was approached.

4. *Report on successes and failures*

‘If we are afraid of failure, then we cannot improve our projects’ (Ellenberger and Richardson 2018: 81). We must have the courage to clearly evaluate and report on the positives and negatives of our project, from all perspectives. This will allow us to improve and ensure mistakes are not repeated.

5. *Seek to understand the methodology behind achieving each outcome*

Evaluation should strive to better understand the relationship between process and satisfactory outcome (Ellenberger and Richardson 2018: 82). Understanding how the various contributions of community archaeology are made by all involved, whether positive or negative, will improve the wider discipline’s theories, methods, and practice (Guilfoyle and Hogg 2015: 5; Heritage Lottery Fund 2019). Additionally, the dialogue required between involved parties to understand the methodology behind each contribution during the project, can improve the ongoing project’s content, design, and delivery, producing a more successful project (McKinnon et al. 2019: 5).

6. *And evaluate in an unbiased fashion*

Community archaeology orients itself towards documenting a more inclusive tale of the past, potentially stripping away biased accounts of history in the process. Evaluations should strive to analyse projects in the most unbiased fashion as possible and seek to mitigate any remaining potential bias (Fowler 2015: 10; Heritage Lottery Fund 2019). Any bias present in the evaluator implicates the validity of the study (Simpson 2009: 117).

## The Methodology

Analysing the aforementioned sources encouraged and informed the development of a new methodology. This methodology strives to address the six ‘needs’ discussed in “What does an evaluation need?” section. The purpose of this evaluation is not to select the best community archaeology projects or methodologies. Every site, community, and culture has different needs. What succeeds in one situation will not necessarily achieve the same results in another. This process instead seeks to identify the various elements of the project that influence its contributions, compare the intended and actual outcomes, and discuss the longevity of these contributions.

### The Framework

The evaluation framework consists of three sections—influencing factors, contributions, and longevity—that identify essential components of the study, highlight project outcomes, and assess the contributions’ sustainability. The influencing factors section lists ten of the most common variables affecting the potential contributions. The second section presents the intended and actual contributions of the project, which frequently differ from one another. This section contains two tables that help evaluate the success of the project against its own objectives for each stakeholder involved. The final section offers a table of prompting questions to address the longevity of the project’s contributions and the principle of the project. The following paragraphs discuss these three sections in detail.

#### Influencing Factors

‘Community archaeology’ and its various sub-categories includes many types of collaborative practice that are ever changing (Atalay 2012: 47). Terms used to label these engagement practices and their definitions vary between regions, nations, time periods, and disciplines. This makes categorizing a project and labelling it difficult, particularly in evaluations. Instead of using labels, the authors chose ten variables that most significantly impact the project’s design, contributions, and longevity. These ‘Influencing Factors’ help categorize the project based on its essential components, rather than labels without clear definitions. This helps reduce confusion in the kinds of community archaeology and their definitions and provides an easy mechanism to compare the core elements of projects.

The Influencing Factors section of the framework consists of a table with three columns: influencing factor, attribute, and description. The ten influencing factors that have been identified are project driver(s), project leader(s), funder(s), participant selection process, location of engagement, nature of engagement, level of engagement, duration of engagement, duration of project, and knowledge sources consulted. The next column, the attributes, lists the most common type of each factor. This allows the influencing factors to be chosen from a list. Pre-defined choices make comparing projects to each other easier. Despite this, the attributes are not rigid. Additional attributes can be added where needed. When not self-explanatory, the adjacent description column further clarifies the attribute. These factors and attributes are not necessarily mutually exclusive, rather a single project could have several attributes listed for each factor. For example, a project’s driver could be a threat to archaeology, academic, and development. All attributes of each influencing factor should be listed. The following paragraphs briefly discuss each influencing factor and how it may affect the project’s contributions. Influencing factors should be analysed individually and alongside other factors when understanding a project’s contributions as they directly impact one another.

##### Project Driver(s)

Project drivers have the potential to influence the project’s composition and contributions through setting pre-determined goals or intentions. Additionally, community engagement today is increasingly required and politically encouraged (Simpson 2008: 4). This may prompt projects to engage the community to ‘tick the box’ for these requirements rather than sensitively engaging the community and considering their needs and wishes (Simpson 2009: 289). For example, a community driver will likely focus on the benefits to the community, whereas a project involving community only as a requirement for funding might not as conscientiously incorporate them. This alters the project’s contributions and the length they endure.

##### Project Leader(s)

A researcher’s own values, needs, and wishes influence the outcomes of research projects (Supernant and Warrick 2014: 581). The project leader therefore can influence the direction of a study, insert biases into the research, or predetermine its outcomes (Simpson 2009: 117; Fowler 2015: 10). For example, an academic project might focus on the theoretical or archaeological contributions of the study, a developer might concentrate on the public relations potential, and a community leader might emphasize community benefit.

##### Funder(s)

Project funders will have their own reasons for funding the project, which can influence the design, potential contributions, and how long the project’s contributions will endure. For example, developer funded community archaeology might engage the public to win community support for a development or to simply meet a specific requirement.

##### Community Participant Selection Process

Community participants may be selected through an application or the opportunity may be publicized through a variety of outlets. This process has the potential to instil biases into which members of the community can participate. In turn, this may positively affect some members of the community while negatively affecting others. For example, a formal application process might exclude illiterate participants or those who do not believe they would be selected, privileging those with self-confidence, literacy, and access to the necessary information and tools to apply. This could cause negative feelings towards the project and in community members themselves who did not apply or who were rejected and feelings of privilege in those selected. Additionally, publicizing the engagement opportunity may exclude members of the community who do not engage with that particular network. For example, opportunities advertised through local archaeological societies would target those already interested in archaeology, whereas radio advertisements will likely reach a wider audience.

##### Location of Site

The site’s physical location can influence how communities engage with the project and exclude or include participants. For example, underwater sites require snorkelling or scuba diving skills to engage with, whereas any able-bodied person can participate with a coastal site. Scuba diving requires expensive certifications and equipment. Lower income communities may be excluded if the engagement only includes this activity, privileging those who can afford it. Furthermore, Simpson (2009) discovered location impacted the learning outcomes achieved in the projects she evaluated. Her study found rural locations met social outcomes more successfully, whereas urban sites more successfully achieved political, economic, and knowledge outcomes (Simpson 2009: 287). This may not be the case across all community archaeology project locations; however, site location is a factor to consider when understanding a project’s contributions and their longevity.

##### Nature of Engagement

The kind of engagement conducted directly influences a project’s potential contributions to each involved stakeholder. For example, the effects community members feel from attending a public lecture on heritage trails will differ significantly from those participating in documenting their elder’s oral histories. Similarly, the researchers giving the public presentation will receive different outcomes than those documenting the oral histories.

##### Level of Community Engagement

As discussed above, community archaeology projects differ tremendously in their methods and levels of community engagement. The level of engagement directly impacts the potential contributions a project can make as participation ultimately comes down to a relationship between power and control (Cornwall 2008: 271). A critical difference exists between going through the motion of public participation without meaning behind the actions and having the power to alter the project (Arnstein 1969: 216).

**Fig. 1:**
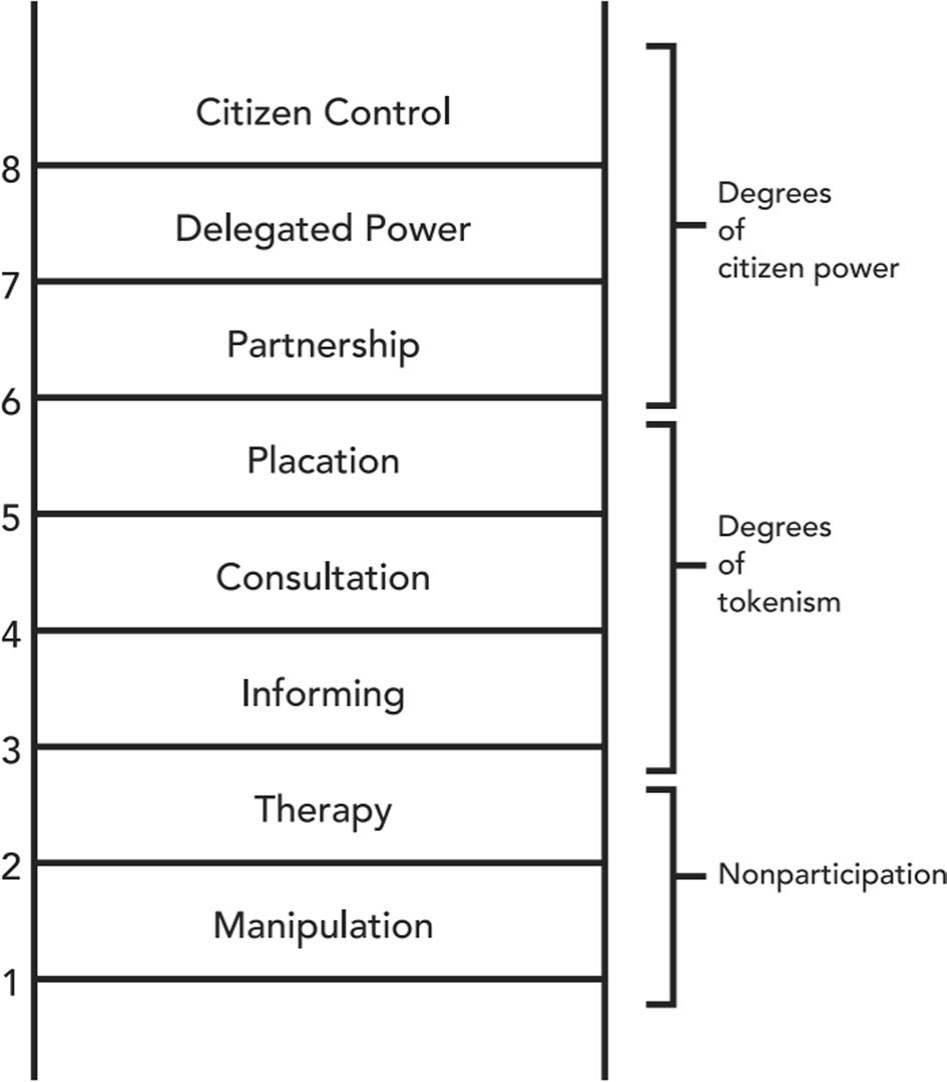
The Ladder of Citizen Engagement (Arnstein 2019:26)

Arnstein (1969) created the ‘Ladder of Citizen Engagement’ to differentiate between these levels of community engagement (Fig. 1). Each rung of the ladder corresponds to a different amount of power allocated to the community (Arnstein 1969: 217). The bottom rungs are levels of non-participation, where traditional powerholders educate the community. At each ascending level of the ladder, more power is transferred from the powerholders to the community. In the middle are degrees of tokenism, where the community can voice their wishes, but lack the power to make decisions. The higher rungs of the ladder include partnerships where communities and those in power, share decision making tasks. The highest rungs of the ladder transfer full power and authority to the community (Arnstein 1969: 217).

Since its initial publication in 1969, scholars have discussed and adapted this ladder to fit a variety of disciplines, including community archaeology as discussed in Cornwall (2008), Belford (2014), Guilfoyle and Hogg (2015), Roberts (2016), and Ellenberger and Richardson (2018). These ladders encompass the flexibility and diversity of community archaeology through encouraging discussions on the balance of power and allowing these dynamics to change (Roberts 2019: 76).

The ladder was adapted to suit this framework and uses the attributes of informing, utilization, consultation, partnership/co-creation, and citizen control (Table 1). The level of engagement indicates how much control and power the community holds. This power level impacts which stakeholder's objectives might be favoured during the project. For example, the informing level favours the powerholders' objectives without input from the community. Projects on this level might not respond to community interests or needs but focus on the powerholder's own.

**Table 1:** The influencing factors of community archaeology projects

Influencing factor	Attribute	Description
Project drivers	Academic	Scholarly research drove the project
	Government	Government requested or prompted project
	Community	Community requested the project
	Development	Archaeology conducted prior to construction
	Funding	The funder required a form of community engagement for funding
	Threat to archaeology	Engagement used to mitigate threats to archaeology from a range of sources including climate change, looting, and as a part of development-driven archaeology
Project leaders	Academic	Universities
	Government	Local, national, or international government
	Cultural resource management (CRM)	Independent archaeology company working hired to manage or research cultural heritage
	Landowner	Legal landowner or tenant
	Heritage organization	Organization involved with heritage
	Community	Non-archaeologists
	Private	Private individual or organization
	Developer	Commercial or other development company
Funder	University	Associated with a university
	Government	Local, national, or international governing bodies
	Non-governmental organization	Charities, trusts, or foundations funded by private individuals without government support
	Developer	Development-driven archaeology where the developer pays for the investigation
Participant selection process	Ancestry or cultural association	Participants discovered via ancestry or cultural association and asked if they would like to participate
	Public advertisement	i.e. TV, radio, and newspaper advertisements both paid for and free
	Community notice	Community advertisements, bulletin board notices, etc.
	Email notice	Emails sent out via address lists or other networks
	Archaeological societies	Archaeology societies help publicize the project
	Application	Formal application process
	Word of mouth	Verbal circulation of the project through established networks
	Walk ups	No pre-selection or notice process, participants simply walked up or asked to participate
Location of engagement	Underwater	Fully submerged
	Intertidal	Partially submerged and exposed due to the tides
	Coastal	In the vicinity of the sea
	Terrestrial	Firmly on land with no locational relation to water
	Riverine	Inside a river or along its banks
	Lacustrine	Inside a lake or along its banks
	Museum	Inside a museum or its collections
	Other built space	i.e. Schools, community spaces, universities
Nature of engagement	Interviews/oral histories	Recording oral histories or community-held knowledge
	Archaeologist led events	Archaeologist created events for community members (i.e. exhibits, workshops, presentations)
	Co-produced events	Events created in collaboration between community members and archaeologists for the community (i.e. exhibits, workshops, presentations)
	Community meetings	Archaeologists meet with the community
	Training sessions	Archaeologists train non-archaeologists in an aspect of archaeological work
	Field school	Archaeologists train non-archaeologists in an academic style in archaeological methods
	Discussion session	Meetings or gatherings where heritage practitioners and community come together to discuss aspects of heritage
	Consultations	Archaeologists asking community member(s) for their advice or expertise in their heritage
	Heritage documentation	Documenting archaeological sites or artifacts
Level of community engagement	Informing	A degree of non-participation where archaeologists or powerholders pass information to the community
	Utilization	Leaders use participants as a source of labour to conduct archaeology (i.e. community volunteers assisting on an excavation) or a source of knowledge (i.e. site locations) without community input into project design, methods, or processes
	Consultation	A degree of tokenism where the community voices their opinions yet lack the power to follow through on them
	Partnership/co-creation	A degree of citizen power where the community can negotiate with power holders and influence the project
	Citizen control	A degree of citizen power where the community has full control. The community consults or employs the archaeologists
Duration of engagement	< 1 day	
	< 1 week	
	< 1 month	
	1–3 months	
	3–6 months	
	> 6 months	
	1 year	
	Multiple years	
Duration of project	< 1 day	
	< 1 week	
	< 1 month	
	1–3 months	
	3–6 months	
	> 6 months	
	1 year	
	Multiple years	
Knowledge sources consulted	Archaeological site	The site itself
	Previous investigations	Reports or other information generated from previous academic or professional investigations
	Published literature	i.e. Books, scholarly articles, blogs, pamphlets, reports, online resources
	Archival information	i.e. Historic documents, photograph collections, public records
	Media	i.e. music (traditional or modern), films, websites
	Cultural knowledge	Belief systems and other knowledge associated with the people who lived near the site
	Legends and myths	Stories passed down from generation to generation
	Community members	I.e. oral histories, stories, memories, place names
	Local archaeologists	Archaeologists who work in the region of the site
	Government officials	People working for government organizations
	Current residents around the site	People who live around the archaeological site today

The ladder is limited as it does not reflect the overall composition of the community and the role of the powerholders. Understanding these relationships are important in successful community archaeology (Roberts 2016: 76). The other influencing factors (Table 1) seek to mitigate this issue. It is important to remember the two groups engaging with the ladder, the community and the powerholders, are not homogenous. Each group contains a variety of viewpoints and interests (Arnstein 1969: 220; Belford 2014: 24).

##### Duration of Engagement

This section refers to the actual length of community engagement, excluding any other project activities. The duration of engagement impacts the potential contributions to all stakeholders involved. For example, shorter durations of engagement may not successfully impart community members with archaeological skills, gather all oral histories of community members, or survey all potential sites (Guilfoyle and Hogg 2015: 27). Conversely, engaging too long or repeating engagement without concrete community benefits risks making the community feel used (Bugumba and Williams 2016: 22).

##### Duration of Project

The project duration refers to the entire length of the project, including all activities that do and do not engage the community. This directly impacts the potential contributions to all stakeholders. Shorter projects present shorter potential time for engagement activities. Longer projects potentially forge deeper bonds between stakeholders and contribute more to each stakeholder. Additionally, shorter projects may prevent community archaeology from having sustainable effects (Simpson 2008: 13; Guilfoyle and Hogg 2015: 27).

##### Knowledge Sources Consulted

Several kinds of knowledge sources exist including books, articles, oral histories, legends, songs, community experts, archaeological data, and more. Different combinations of these knowledge sets will influence the project’s findings, through having the potential to privilege particular accounts of the past, omit aspects of history, or disregard community-held knowledge amongst other potential influences (Atalay 2012: 75; Chirikure and Pwiti 2008: 474; Supernant and Warrick 2014: 566). This influencing factor indicates which knowledge sources were consulted, stimulating a discussion on how the sources may impact the results. For example, consulting published literature and archival information alone privileges written accounts of the past and preserves any biases or omissions found within these sources. This could omit the inclusion of intangible heritage or perpetuate one-sided accounts of history. Conversely, consulting oral histories and cultural knowledge alone includes intangible community-held knowledge, contributing to the preservation of their culture and recognizing them as an authority on their past (Chirikure and Pwiti 2008: 467; Ford 2011: 1). Privileging these accounts of the past over others may lead some to disregard academically discovered knowledge that claims another version of history.

#### Contributions

The contributions section of the framework consists of two tables (Tables 2 and 3) outlining a project’s intended and actual contributions for involved stakeholders. Intended contributions are outcomes, outputs, or goals project leaders or participants determine at the beginning of a project. The actual contributions are those achieved or noted at the end of the project. Contributions may be positive or negative. Both should be reported in the tables. Comparing these two tables helps evaluate a project’s successfulness in achieving its own goals. Using this in combination with the influencing factors, will help archaeologists understand the correlation between them. This information could be used in designing and carrying out more successful, positive projects.

**Table 2:** Intended contributions. The contributions column is blank and should be filled in with positive and negative contributions when used in an evaluation.

Beneficiary	Category	Contribution
Community	Cultural	
	Social	
	Economic	
	Educational	
Academic	Theoretical	
	Methodological	
	Knowledge gained	
Heritage	Management	
	Impact on the archaeology	
	Decolonization of history

**Table 3:** Actual contributions. The contributions column is blank and should be filled in with positive and negative contributions when used in an evaluation.

Beneficiary	Category	Contribution
Community	Cultural	
	Social	
	Economic	
	Educational	
Academic	Theoretical	
	Methodological	
	Knowledge gained	
Heritage	Management	
	Impact on the archaeology	
	Decolonization of history	

Both the intended and actual contribution tables follow the same structure. The first column outlines project stakeholders. The most common stakeholders, and thereby beneficiaries of the project, fall into three categories: community, academic, and heritage. Other beneficiaries may exist and can be added to this framework where necessary. The second column, labelled category, presents the common areas where the beneficiaries receive contributions. For example, the community may be affected culturally, socially, economically, or educationally. These are therefore the categories for the community beneficiary. The last column is blank. It should be filled in with a list of the project’s positive and negative contributions in the appropriate row. Where a beneficiary or category is irrelevant for the project, it should be deleted. Tables 2 and 3 list the beneficiaries, categories, and leave the contributions section blank.

#### Longevity

Project leaders rarely publicly discuss the intended or actual longevity of their program’s contributions, making analysing this area challenging. Some regrettably, are inclined to take an approach known as ‘parachuting’ where leaders literally drop into a community, conduct their research and leave with no intention of ever generating a sustainable legacy. Thus, community archaeology projects may or may not intend to cause long-lasting effects. Despite this, the sustainability of the work’s contributions, both positive and negative, should strive to be understood. The last section of the framework (Table 4) lists four closed-ended questions that prompt a discussion of the longevity of the projects’ contributions. The answer in the second column may be yes, no, or unknown. The following paragraphs list the questions in Table 4 with a brief description of the kinds of discussions the questions could stimulate.

**Table 4:** Longevity of the project's contributions. See “Longevity” section for a discussion on their use

Question	Answer
Is there planned or continued engagement after project completion?	Yes
	No
	Unknown
Is there new or continued research after the project is finished?	Yes
	No
	Unknown
Is the research publicly accessible?	Yes
	No
	Unknown
Is there continuity in the principle of the project?	Yes
	No
	Unknown

#### Is there planned or continued engagement after project completion?

Planned or continued engagement would indicate whether the direct contributions of the engagement process will continue. For example, if community members are paid for their time, a ‘yes’ would prompt a discussion of how the economic contributions to the community would continue. A ‘no’ might signal an end to these contributions.

#### Is there new or continued research after the project is finished?

New or continued research would encourage a discussion of the future research and help highlight the longevity of associated contributions. No further research indicates a shorter longevity of these contributions.

#### Is the research publicly accessible?

Publicly accessible research would allow the community and academia to continue learning from the study. Additionally, this could impact the longevity of heritage’s decolonization or management into the future. If research was not publicly accessible this would shorten the longevity of the more educational contributions of the project.

#### Is there continuity in the principle of the project?

This question seeks to stimulate a discussion of whether the point of the project will endure. For example, if the purpose of the project is to decolonize history, are there mechanisms in place to ensure the alternative accounts of history are shared? Conversely if the purpose of the project is to build a network of volunteers to document at risk sites on their own, do these participants have the skills and confidence to continue recording after the project finishes? Furthermore, will they?

### Using the Framework

This framework functions as a flowchart. Each table (Tables 1, 2, 3, and 4) should be filled out and synthesized in a discussion. To ease discussion and comparing projects to one another, Table 1 should be filled out using the attributes listed in this table. If none pertains, additional attributes can be added. Table 2 and 3 present the most common beneficiaries of the project. Others can be added as well as additional relevant categories. The ‘contribution’ column should be populated with the project’s intended (Table 2) and actual contributions (Table 3) for each relevant beneficiary and category. The beneficiaries and categories that do not apply to the investigation should be deleted. Removing the irrelevant sections makes reading the table and viewing the pertinent information easier.

## Case Studies

As a part of a larger research project, five case studies were selected from a large pool of potential projects to demonstrate the use of this framework to analyse projects retrospectively. Three of these case studies are discussed in the following sections. Each project is analysed against itself, asking how the project contributed to the community, academia and heritage, as well as the longevity of these outcomes using publicly available information. These projects were selected as they meet three requirements. Each case study engages with maritime or underwater cultural heritage, self-identifies as a ‘community archaeology’ project, and provides publications, webpages, or other easily accessible sources of information about the project. The case studies discuss projects from both developed and developing countries. Publicly available information, such as academic articles, websites, and social media, and conversations with a leader of each project, helped inform these evaluations. As a collection, these case studies present projects with differing influencing factors, particularly the level of community engagement. This helps illustrate the framework’s suitability for analysing the wide range of projects within the community archaeology category.

Due to the time constraints of this research project, case studies were analysed retrospectively, either near the end or after the project finished. All components of the evaluation, including the intended contributions tables, were filled out using publicly available resources, funding applications, or from conversations with project leaders. Case studies therefore only demonstrate the framework retrospectively. However, in theory this framework could or even better should, be used when designing community archaeology projects, or at any point during the course of the project. Additionally, the framework can also be used by the communities themselves, as well as funders and other stakeholders.

The three case studies are War in the Pacific: A Difficult Heritage, Bahari Yetu, Urithi Wetu, and the Coastal & Intertidal Zone Archaeological Network (CITiZAN). The authors have no professional connection to War in the Pacific: A Difficult Heritage. Blue is a co-investigator on Bahari Yetu, Urithi Wetu and Bell participated on the fieldwork conducted in March 2019. For this case study, sources include those stated and personal experience. Bell also participated on CITiZAN fieldwork in connection with the University of Southampton in Autumn 2019. Sources for the CITiZAN evaluation, however, rely exclusively on sources stated.

### War in the Pacific: A Difficult Heritage

The National Endowment for the Humanities, an independent United States federal organization, funded *War in the Pacific: A Difficult Heritage*, https://cnmiheritage.wordpress.com; a self-identified community archaeology project in Saipan. Other partners included East Carolina University (ECU), the Northern Marianas Humanities Council (NMHC), Veterans Affairs Office, and the Historic Preservation Office (CNMI Heritage 2019; McKinnon et al. 2019: 2). McKinnon, Ticknor and Froula identify this work as a community and indigenous archaeology project (McKinnon et al. 2019: 2).

This project sought to engage a community of Pacific Islander veterans and families in two ways. First, the program trained members of the Saipan community with experience in humanities and veterans affairs as Discussion Leaders. The trainees were largely self-selected or previously worked with McKinnon. Discussion Leaders completed a 1-week training program, conducted by the ECU Project Directors, to refine their skills in asking open-ended questions, listening without critique, and encouraging openness and trust (McKinnon et al. 2019: 5). The training prepared the Discussion Leaders to assist in leading the second engagement activity.

The second form of engagement, the Discussion Program, engaged veterans, surviving civilians, and associated family members of World War II (WWII). The trained Discussion Leaders helped guide each discussion program. The program centred around the Spanish-Chamorro Wars of the seventeenth century and the WWII Battle of Saipan. These wars represent the ‘bookends to the history of resistance and aggression in the islands’ (McKinnon et al. 2019: 5). The first Discussion Program lasted one week. After feedback from participants, project leaders extended the second program to last two weeks (McKinnon 2017b: 1). The program consisted of discussion sessions to explore war-related humanities resources each weekday and visits to archaeological sites on the weekend. Themes included Veteran/Warrior and the Indigenous identity, the Enemy, the Civilian, Memorialization, and Conflict Heritage (McKinnon et al. 2019: 6). The program sought to explore conflict as a shared cultural heritage and human experience, provide opportunities for generational knowledge sharing, and re-integrate Pacific Islander veterans into a socio-cultural position of authority on war in their islands. At the conclusion of the program, participants received a certificate and a challenge coin designed for the program. Challenge coins hold significance to veterans and military families, signifying an achievement, success, or special effort in service (McKinnon et al. 2019: 5).

In addition, the project also uploaded Discussion Program materials to their website, in a password protected area, to enable future access to the documents and continue the discussion. The project also conducted a program evaluation consisting of surveys taken before and after each discussion session (McKinnon 2018: 3). On-island engagement with the community lasted 4 weeks:  week for Discussion Leader training and 3 weeks for the two Discussion Programs. The grant period lasted approximately one year from May 2017–July 2018 (National Endowment for the Humanities 2020). The level of engagement rests at Partnership/Co-Creation as Project Leaders were actively engaged with the community from the inception of the grant idea through to today with future projects that evolved out of War in the Pacific. Team members actively listened to community concerns and feedback, adapting the project accordingly. Furthermore, the nature of engagement lent itself to open discussions between all participants, regardless of stakeholder group.

#### Contributions

The Discussion Leader and Discussion Programs succeeded in exploring conflict and war as a shared human experience (McKinnon et al. 2019: 9). The only intended contribution that may not have been met is the decolonization of history, specifically better understanding the Spanish-Chamorro Wars. The publications and resources available to date do not discuss this. Future papers may cover this topic. The factors outlined in Table 5 influenced the contributions of the project.

**Table 5:** Influencing factors in War in the Pacific (Information from McKinnon 2018; CNMI Heritage 2019; McKinnon et al. 2019)

Influencing factor	Attribute
Project drivers	Academic
Project leaders	Academic
Funder	Government
Participant selection process	Public advertisement
	Community notice
	Email notice
	Word of mouth
Location of engagement	Coastal
	Underwater
	Other built space
Nature of engagement	Training sessions
	Discussion sessions
	Site tours
Level of community engagement	Partnership/co-creation
Duration of engagement	< 1 month (all on-island engagement activities)
Duration of project	1 year
Knowledge sources consulted	Published literature
	Media
	Cultural knowledge
	Community members

The project leaders planned to engage 10–15 people per discussion session program, totalling 20–30 people. However, over 60 people participated in the two discussion sessions with an additional 30 attending the public film viewings (McKinnon 2018: 2; McKinnon et al. 2019: 7). Available information did not indicate a shift in the design or running of the Discussion Sessions due to the larger than anticipated number of participants. Several factors could have influenced the larger than anticipated number of participants. McKinnon previously worked on community archaeology projects in Saipan. A positive reputation may have preceded her, or she could have utilized established networks to reach a wider audience. Funding from both national and local government organizations may impact the networks reached as well, particularly the Veterans Affairs Office. Additionally, the opportunity was publicized through a wide range of sources including TV, radio, newspaper interviews, paid advertisements, fliers, emails, and word of mouth (McKinnon et al. 2019: 7). The diversity in advertisement methods meant a large number of people could be reached. Finally, the level of community engagement might have affected the number of participants. Members of the Saipan community were trained as Discussion Leaders, ultimately leading the program alongside ECU project directors. Discussion Leaders may have reached out to their own networks for potential participants and encouraged those wary or shy of programs run by outsiders to feel more comfortable. During the program, this also might have allowed Discussion Leaders to engage with participants on a cultural level (Tables 6, 7 and 8).

**Table 6:** Articulated intended contributions of War in the Pacific (Information from McKinnon 2018; CNMI Heritage 2019; McKinnon et al. 2019)

Beneficiary	Category	Contribution
Community	Cultural	Explore conflict as shared cultural heritage
		Provide opportunity for generational knowledge transmission
		Present participants with a challenge coin as a token of completion
	Social	Re-integrate Pacific Islander veterans into a sociocultural position of authority regarding the history of war on their islands
	Economic	Pay discussion leaders for both the training sessions and the discussion sessions
	Educational	Help the community gain a meaningful, relevant understanding of war as a shared human experience
		Provide discussion materials in print and online
Heritage	Decolonization of history	Understand the under studied Spanish-Chamorro Wars, specifically how indigenous people resisted and negotiated with colonial powers

**Table 7:** Articulated actual contributions of War in the Pacific (Information from McKinnon 2018; CNMI Heritage 2019; McKinnon et al. 2019)

Beneficiary	Category	Contribution
Community	Cultural	Explored local conflict as shared heritage
		Shared untold stories, even amongst friends
		Presented participants with a challenge coin as a token of completion
	Social	Prompted words of thanks between veterans of previous wars and current service men
		Shed tears for those who were lost
		Therapeutic or restorative benefits to participants
	Economic	Paid discussion leaders for training and discussion sessions
	Educational	Recognized war is universal and stretches from the past into the present, invoking a stronger appreciation for the heritage representing those wars
		Discussion leaders were trained in reading texts and leading discussion sessions. Survey data reflected this success
		Provided print and online discussion materials
Academic	Methodological	Created methods and content for discussion programs that could be replicated
	Knowledge Gained	Presented project at academic conferences and ECU events
		Earned an award from ECU
Heritage	Impact on Archaeology	Participants developed a stronger appreciation of the physical remains of the wars
		The improved understanding and awareness of the archaeological resources likely improved the stewardship of these spaces

**Table 8:** Longevity of the contributions of War in the Pacific

Question	Answer
Is there planned or continued engagement after project completion?	No
Is there new or continued research after the project is finished?	Yes
Is the research publicly accessible?	Yes
Is there continuity in the principle of the project?	Unknown

Project leaders did alter the duration of the second program in response to feedback from the first Discussion Session. The first discussion program lasted one week. Due to feedback from the first, project leaders extended the program by a week to two weeks long, allowing for more time to go through the materials (McKinnon 2018: 12). Project leaders also altered the program for three minors that participated. Rather than preventing them from participating, leaders adapted the program by limiting the minors’ fieldtrips to terrestrial sites only (due to ECU’s policy on boats) and requiring parent permission (McKinnon, 2018: 12). All participants came from the islands of Saipan and neighbouring Tinian. Participants were aged 13–85 with a 1:1 ratio of men to women (McKinnon 2018: 2).

During the week-long Discussion Program, participants were asked to read or watch accompanying materials before each session. These materials included articles, websites, poems, books, films, site plans, and public service announcements (CNMI Heritage 2019). This wide range of knowledge sources, in addition to the participant’s own memories and familial histories, presents various perspectives and experiences of the wars, deepening everyone’s understanding. This may have also impacted their site visits.

An unintended outcome for both heritage and the community, was the reconnection and stronger appreciation of the physical remains left from the wars their ancestors, families, and even themselves participated in (McKinnon et al. 2019: 9). After five days of discussing the islands’ wars in depth, the participants visited heritage sites in coastal and underwater environments assessable on foot or using snorkelling equipment (McKinnon et al. 2019: 5). Discussing the history and lived experiences of the wars before visiting the sites likely brought the facts, stories, and memories about the places into the forefront of their minds, making the visits even more impactful. Despite growing up and presently living on the island, some of the participants never before visited the selected heritage sites. Visiting these places invoked strong reactions, particularly to the civilian caves. Civilians used caves as shelter during the war. Remains of their habitation exist today as well as small individual or family offerings of food, memorials, or personal objects (McKinnon 2015: 148; McKinnon et al. 2019: 8). Participants may have strongly reacted to these places as they likely grew up hearing stories of their own family members living in these caves during the wars (McKinnon et al. 2019: 9). Although the programs did not intend to have therapeutic or restorative benefits, the results seen in person and in survey results were evident (McKinnon 2018: 9).

#### Longevity

##### Community and Heritage

The program is currently being evaluated through surveys collected prior to and during the program itself (McKinnon et al. 2019: 7). Preliminary data portrayed an overwhelmingly positive response (McKinnon 2017: 3). Further survey content and results are not publicly available at this time. The synthesized survey results will be turned over to the Northern Marianas Humanities Council (NMHC) for review and to determine whether the project will continue in some capacity (McKinnon et al. 2019: 8). No additional engagement is scheduled (CNMI Heritage 2019). ECU program leaders intend to further publish and reflect on this project (McKinnon et al. 2019: 8). This information would add to the discussion of the project’s contributions and potentially illuminate the longevity of these outcomes to community and heritage beneficiaries. Project leaders applied to three further grants to continue related work (McKinnon 2018: 11). Two of these bids were unsuccessful. The National Endowment for the Humanities (NEH) awarded the third. This project, on hold due to COVID-19, will engage teachers in conflict heritage utilizing content developed out of War in the Pacific.

The present research and resources used in the Discussion Program are publicly available online. A password protected area is available for both Discussion Leaders and program participants. It is unknown what resources or information may be available there. Project leaders hope the website and online platform would encourage sustainability of the program and encourage continued access (McKinnon 2017a: 1).

Due to the intense nature of the topic, the contributions to the community culturally, socially, and educationally will likely continue, particularly when visiting or passing war related heritage sites. If so, there would be continuity in the principle of the project. The challenge coin and certificate participants received, may also serve as an additional reminder of the experience and knowledge gained. This will likely hold true for project leaders as well. McKinnon stated what she learned will continue to impact her and her work beyond the end of the program (McKinnon 2020, personal communication, 11 August).

##### Academic

The methods employed and content created for both the Discussion Leader workshop and Discussion Program could be re-used in future programming (McKinnon 2018: 10). Academic contributions also consisted of presenting the project at conferences and ECU events, helping educate listeners on the project, its effects, and methods employed. Additionally, the project earned two awards for scholarship engagement and community engagement (McKinnon 2018: 13). War in the Pacific also helped earn funding for the aforementioned NEH project due to run in 2021, COVID-19 dependent.

### Bahari Yetu, Urithi Wetu

Tanzania’s coasts host a unique, living maritime cultural heritage. Yet the communities at the heart of this living heritage face a range of threats, including overfishing, development, and supply-chain disruptions. At the same time, tourism and social media threaten and exploit this heritage without benefiting local communities either socially or economically. *Bahari Yetu, Urithi Wetu*, https://risingfromthedepths.com/bahari-yetu-urithi-wetu/ (Our Ocean, Our Heritage) sought to understand the maritime cultural heritage of Bagamoyo and Milingotini, a coastal fishing town and village respectively in Tanzania, and how this heritage could be harnessed to benefit their own communities. This project ran in partnership between the University of Exeter, University of Dar Es Salaam (UDSM), and University of Southampton. The Global Challenges Research Fund (GCRF) *Rising From the Depths* programme funded the project.

Functioning on the Partnership/Co-Creation level of engagement, Bahari Yetu, Urithi Wetu (BYUW) sought to understand the context of the maritime cultural heritage of the coastal communities of Bagamoyo and Milingotini, what they define as important, and what threatens it through ethnographic, community-based approaches (Cooper et al. 2019: 2). Ichumabaki previously conducted years of research along the Bagamoyo and Milingotini coastline, forging strong relationships with community members and gatekeepers. This helped BYUW research begin easily and access essential community members. Methods included conducting interviews, attending and creating community events, and hosting workshops and discussion sessions. This project documents the tangible heritage in the form of boats and fishing gear, their construction and use, and the associated intangible heritage (Cooper et al. 2019: 2). Students from UDSM learned how to document maritime heritage and helped conduct the fieldwork (Cooper et al. 2019: 3). A community-based exhibition was co-created and presented information learned from the project. Local children, community members, tourists, and USDM and government officials visited the exhibition. The project also helped establish a boatbuilders association, called CHAMABOMA-Bagamoyo, for the community to perpetuate their heritage, give the boatbuilders themselves a voice, and provide a means for skills training, boat building and tourist engagement (Cooper et al. 2019: 3).

The project was planned to run from June 2019–June 2020 (Cooper et al. 2019: 3). The project began with an initial co-creation workshop. This workshop included members of the fishing, inter-tidal gathering, and boat building communities and the BYUW researchers. Together they laid out objectives and goals of the project. Two field seasons followed: August 2019 and March 2020. Due to the unforeseen circumstances of COVID-19, the project required an extension for the remaining activities. These activities included a maritime heritage week at USDM consisting of a re-run of the exhibition previously held in Bagamoyo, the launch of the BYUW music video and a documentary of building a *ngalawa*, and culminating in a maritime heritage stakeholder meeting involving stakeholders from government agencies, the Bagamoyo boatbuilding and fishing community, and academics. This paper discusses the work completed up to September 2020 (Tables 9, 10, 11 and 12).

**Table 9:** Influencing factors of BYUW (Cooper et al. 2019)

Influencing factor	Attribute
Project driver	Threat to archaeology (main driver)
	Funding
	Academic
Project leader	University
Funder	Governmental organization
Participant selection process	Word of mouth
	Walk ups
Location of engagement	Coastal
Nature of engagement	Interviews/oral histories
	Co-produced events
	Community meetings
	Training sessions
	Discussion sessions
	Heritage documentation
Level of community engagement	Partnership/co-creation
Duration of engagement	One year
Duration of project	One year
Knowledge sources consulted	Archaeological site
	Published literature
	Archival information
	Media
	Cultural knowledge
	Community members
	Government officials
	Current residents around the site

**Table 10:** Intended contributions of BYUW (Cooper et al. 2019)

Beneficiary	Category	Contribution
Community	Cultural	Create an organization to facilitate the longevity of local boatbuilding traditions and a location for community-based educational resources
		Engage with the community to document the maritime cultural heritage of the place, helping understand the value and priority of maritime practice and connections
	Economic	Develop connections with neighbouring tourist destinations and promote Bagamoyo as a tourist destination for maritime cultural heritage
		Promote selling boat models and maritime tours on the boats to tourists
		Create an organization to facilitate the longevity of local boatbuilding traditions and generate economic benefit for the community
	Educational	Develop and install a temporary, small scale exhibition for the local community and tourists
		Train students and early career researchers from UDSM in community-based fieldwork
		Raise awareness about the value and significance of maritime cultural heritage
Academic	Methodological	Commission the build of a *ngalawa* and record the building process
		Undertake documentation and ethnographic enquiry
	Knowledge gained	Publish findings in relevant international maritime or heritage journals, communicating the project’s findings
Heritage	Management	Share the community’s perspectives with local and international stakeholders and what challenges and threats face their maritime cultural practices

**Table 11:** Actual contributions of BYUW (Cooper et al. 2019)

Beneficiary	Category	Contribution
Community	Cultural	Documented the tangible and intangible maritime cultural heritage of Bagamoyo and Milingotini
		Mapped place names and locations of various maritime cultural activities (i.e. fishing, boat storage, boatbuilding, and gathering locations)
	Social	Founded CHAMABOMA-Bagamoyo, joining together crafters and enhancing an already present sense of community
		Improved relationships between the fishermen, boatbuilders, and community members
	Economic	CHAMABOMA-Bagamoyo provides a framework for earning money through the association through offering boat rides
		Secured promises from officials for additional funding for CHAMABOMA-Bagamoyo
		Surveyed tourists to determine what they wanted to experience as a part of the maritime cultural heritage
	Educational	Produced an exhibition for the community and tourists
		Created a documentary of the making of a *Ngalawa*
		Trained UDSM students and early-career researchers in data collection and provided fieldwork experience
Academic	Methodological	Commissioned a *ngalawa* and documented the entire process of building this kind of vessel
	Knowledge gained	Intends to publish several academic articles on the findings, sharing information on a historically understudied area
Heritage	Management	Improved communications between the community and the government as well as provided avenues for continued discussions
	Decolonization of history	Improved understanding of fishing and boatbuilding practices in an understudied area of the globe

**Table 12:** Longevity of BYUW

Question	Answer
Is there planned or continued engagement after project completion?	Yes
Is there new or continued research after the project is finished?	No
Is the research publicly accessible?	Yes
Is there continuity in the principle of the project?	Yes

#### Contributions

To date BYUW achieved all of its intended contributions as well as a few unintended contributions. The project’s inclusive design and collaboration between Tanzanian and international researchers impacted these results. University participants consisted of those from the UK and Tanzania. Tanzanian researchers helped bridge cultural and linguistic boundaries for UK participants especially as they had already established contacts in the region. In turn, participants may have been more willing to converse with researchers due to the presence of Tanzanians, thereby positively affecting the information gained from data collection. University project leaders likely lead to the contribution of student and early career researcher training and the quantity of academic articles intended to be published.

The location, nature, and level of engagement, participant selection process, and knowledge sources consulted, encouraged gathering both tangible and intangible data from a variety of users of the coast. Participants included boatbuilders, cargo-ship workers, boat captains, small and large boat fishermen, sea cucumber gatherers, nail makers, and spiritual practitioners, amongst others. Potential participants were contacted either through established networks from Ichumabaki’s previous work, identified through the initial co-creation workshop, or approached on the beach as they engaged in fishing, boatbuilding, gathering, or similar activities. This allowed a range of people who engage with the maritime space to be included. However, the daily duration of engagement occurred for a relatively short period of time during daylight hours, potentially excluding those who fished at night if they were not on the beach during daylight hours. The variety of knowledge sources consulted, and diverse array of people engaged with helped establish a well-rounded understanding of the maritime culture present. Consulting oral histories and cultural knowledge in addition to archival information, provided a more complete picture than if archival or written information had been used exclusively, particularly as written information about the maritime heritage of this area is scarce. The level of community engagement instilled in participants a sense of pride and ownership of their heritage and also the project. This is evidenced through the boatbuilders association changing their name from MMCHA, what the project leaders had selected, to CHAMABOMA-Bagamoyo. CHAMABOMA-Bagamoyo means the Association of Boat Builders and Vocational Training. This aligns more with their hopes for the organization and resonates more with the boatbuilders. Engagement with a core group of informants guided the study and significantly impacted the produced exhibition. Volunteers from the community, who were also informants in the project, donated personal items for the exhibition and acted as guides, providing first-hand information to visitors about the role they play in the maritime cultural landscape. They lead the exhibition once the doors opened and made real connection with local, out-of-town, and overseas visitors.

The project’s unexpected contributions included data gathered from mapping of place names and most frequently visited fishing spots as well as quantitative survey data from tourists about their visit to Bagamoyo and what would make it more enjoyable. Producing the exhibition brought together boatbuilders, fishermen, and community members. This led to the unexpected contribution of improving social relationships between these groups who did not otherwise necessarily mix. Unintended contributions also consisted of producing a documentary film of building a *ngalawa*, helping share the in-depth process to wider audiences. In addition, the exhibition was not anticipated to directly reach high levels of the Tanzanian government, SADC culture ministers and permanent secretaries, and USDM Officials. SADC culture ministers and permanent secretaries happened to be in Bagamoyo during the exhibition and attended it. These connections forged have the potential to significantly positively impact the local people.

#### Longevity

At this time, the UK directors do not have further engagement planned for after the project’s completion. They aspire to continue their work with additional funding. However, Ichumabaki will likely continue research alongside this community in related future projects, therefore the answer to the first two questions are conditionally yes. Without Ichumabaki’s likely further research and without funding to continue BYUW, the answer to the first two questions would be no. CHAMABOMA-Bagamoyo members are keen on continuing their organization; however, additional financial support is needed to help the organization be self-sufficient. Funds were secured to purchase an engine for the *ngalawa* built for CHAMABOMA-Bagamoyo. This will allow CHAMABOMA-Bagamoyo to take out tourists regardless of the winds and conduct a safer operation, hopefully improving the organization’s financial security.

##### Community

The project and exhibition helped showcase the community and their cultural heritage to the community itself. The exhibition helped communicate information to locals and visitors previously unaware of the rich maritime culture of the place. School trips helped educate children on the heritage and industry of the area. Benefits to this include instilling more of a sense of place and connection to the sea; despite living near the ocean they can experience sea blindness. In addition, visiting the exhibition may pique their interest in becoming boatbuilders or fishermen themselves. Several project participants commented on how the exhibition and CHAMABOMA-Bagamoyo instilled in them a sense of pride in their own industries and heritage. This pride hopefully will endure alongside new friendships gained and a greater sense of purpose in their work.

##### Academic and Heritage

The research will be accessible through a number of academic journals intended to be published. Project directors intend to publish open-access where possible. This will help ensure the information gained will be accessible and shared with anyone with internet access. The documentary film produced will also aide this and perpetuate the traditional process of building a *ngalawa.* This resource is vital should boatbuilder numbers dwindle and youth do not necessarily want to perpetuate the craft.

The networks and connections established at the exhibition between community, academics, and government officials will likely endure and have significant impact on the local community. UDSM has already promised significant funds for the development of a maritime museum in Bagamoyo and for the continued running of CHAMABOMA-Bagamoyo.

The principle of the project is to document maritime cultural heritage and understand threats facing these communities and their heritage. The information gained and shared will ensure the information continues to reach audiences. If the connections forged and CHAMABOMA-Bagamoyo endure, additional goals of providing mechanisms to perpetuate the heritage of the area and reap benefits for the local communities will occur. The project also sought to raise visibility and awareness of what the community values about their own heritage. If the pending policy workshop and government relationships continue, these contributions will continue. If these contributions end, the principle of the project will draw to a close as its short-term nature does not lend itself to develop a significant long-lasting impact.

### Coastal and Intertidal Zone Archaeological Network (CITiZAN)

Funding for the England based *CITiZAN*, https://citizan.org.uk project came from a Heritage Lottery Fund (HLF) grant with match funding from the National Trust and the Crown Estate. The grant ran from January 2015 to June 2018. Partners included the Council for British Archaeology and the Nautical Archaeology Society. The Museum of London Archaeology (MOLA) hosted the project (Ostrich et al. 2018: i). A new grant has been secured for additional programming, called CITIZAN 2019+. This analysis and discussion will only cover the 2015–2018 project.

England hosts a wealth of archaeological sites in the coastal and estuarine environments, from prehistoric settlements to World War Two sites. Coastal erosion and sea-level rise threaten these sites. CITiZAN was created to establish a standardized methodology to record these sites across the English coastline and create a network of volunteers to record, monitor, and promote the coastal archaeology (CITiZAN 2019). CITiZAN hosted events included guided walks, lectures about coastal heritage and monitoring work, training events, and more. CITiZAN also hosted two placement students (Ostrich et al. 2018: 18).

As discussed in “How do we evaluate?” section, HLF funded projects require an evaluation. CITiZAN used participant feedback forms and individual and group interviews throughout the program as a mode for assessment. Esther Gill from Bright Culture conducted an evaluation at the end of each year and compiled a final summative evaluation report. Each year’s evaluation focused on a different element of the project: outreach and training programme (2015), *the smartphone App* recording system (2016) and engaging young people (2017) (Ostrich et al. 2018: 31). These evaluations can be found online. Aspects of each evaluation are discussed below (Tables 13, 14, 15 and 16).

**Table 13:** Influencing factors of CITiZAN (Information from Gill 2017, 2018; Ostrich et al. 2018; CITiZAN 2019)

Influencing factor	Attribute
Project driver	Threat to archaeology
Project leader	Heritage organization
Funder	Government
Participant selection process	Word of mouth
	Local archaeological societies
Location of engagement	Intertidal
Nature of engagement	Archaeologist led events
	Training sessions
	Heritage documentation
Level of community engagement	Utilization
Duration of engagement	< 1 Day (each engagement)
Duration of project	Multiple years
Knowledge sources consulted	Archaeological site
	Archival information
	Community members
	Current residents around the site

**Table 14:** Articulated intended contributions of CITiZAN (Information from Gill 2017, 2018; Ostrich et al. 2018)

Beneficiary	Category	Contribution
Community	Social	Create an enjoyable social experience and promote well-being through active engagement
	Educational	Develop and implement training sessions on recognizing and recording archaeological features
		Directly engage new audiences in their heritage through lectures, training workshops, coastal walks and other activities
		Encourage and support communities to become active partners in research, writing, and sharing of heritage
		Create an open-access interactive App and website for recording and viewing community-sourced data on England’s coastal heritage
Academic	Methodological	Create, promote, and teach a standardized survey and monitoring methodology for coastal heritage sustainable beyond the program
	Knowledge gained	Establish a network of volunteers trained to record, monitor, and promote threatened coastal sites
		Document and disseminate findings through reports and the Archaeological Data Service, encouraging further research, analysis, and interpretation on a larger scale
		Organize an annual conference
Heritage	Management	Establish a national system for documentation, leading to improved monitoring and management of coastal heritage
	Impact on archaeology	Raise awareness of at-risk coastal and intertidal sites

**Table 15:** Articulated actual contributions of CITiZAN (Information from Gill 2017, 2018; Ostrich et al. 2018)

Beneficiary	Category	Contribution
Community	Social	Offered an opportunity for people to meet and engage with others and make friends on an enjoyable day out
	Economic	Boosted local economies by hiring local venues, catering facilities, and accommodations for conferences
	Educational	Held 243 outreach events, directly engaging 9234 people
		Carried out 120 training events with 1337 people attending and developing skills. 583 individual trainees, many of whom have returned for multiple sessions
		Developed a virtual community with 1609 Facebook likes, 2273 Twitter followers, 220 Instagram followers, 2352 email subscribers, and 2535 registered CITiZAN App users with nearly 58,000 individuals viewing the website
		Educated about the heritage that surrounds the public everyday
		Shared knowledge about climate change and coastal erosion
Academic	Methodological	Created and implemented a standard of recording England's threatened coastal heritage
	Knowledge gained	Established a network of local communities and archaeologists
		Added 2527 new features to the interactive coastal map, 2289 monitoring updates, and 3927 new photos of heritage assets. All deposited with the Archaeological Data Service and local and national bodies
		Held three national conferences
		Delivered papers and sessions at local, national, and international conferences
		Produced 23 grey literature reports of key sites that were delivered to Archaeological Data Service and Historic Environment Record offices, and available on the website
Heritage	Management	Produced an interactive website and smartphone App and monitoring scheme that helps highlight the state and importance of individual sites, potentially leading to improved site management
	Impact on archaeology	Raise awareness of at-risk coastal and intertidal sites

**Table 16:** Longevity of CITiZAN's contributions

Question	Answer
Is there planned or continued engagement after project completion?	Yes
Is there new or continued research the project is finished?	Yes
Is the research publicly accessible?	Yes
Is there continuity in the principle of the project?	Yes (with funding)

#### Contributions

CITiZAN achieved its intended outcomes as well as a few unintended outcomes. The project driver, threat to archaeology, instils a sense of urgency and sense of purpose in participants. Evaluations revealed community benefits of providing an opportunity to engage actively, meet people, and feel like they are making a difference (Gill 2018). This is likely due to the project driver and nature of engagement, bringing together a group of people to document an archaeological site with the intent of preserving the information for future generations.

The level, nature, and duration of engagement allowed CITiZAN to reach a large audience in three years. The intertidal environment further encouraged a large number of participants as it provides a relatively easily accessible location, depending on the volunteers’ ableness and the intertidal terrain. Additionally, the strong message regarding climate and coastal change threatening heritage drew in participants. The involvement of CITiZAN team members on ‘Britain at Low Tide’, a TV show discussing the archaeology present at low tide, helped draw in participants. CITiZAN’s evaluations indicate outreach events directly engaged 9234 people, while 1337 people attended training sessions. Of those attending training sessions, 583 individuals attended the events, with some individuals taking part in more than one session (Ostrich et al*.* 2018: 31). It is unclear exactly how many of the participants repeated training sessions or how many went on to record sites on their own.

One unintended outcome presented itself in the economic contributions to the local community. Conferences boosted the local economy through hiring local venues, catering facilities, and accommodations (Ostrich et al*.* 2018: 70). Similar economic contributions may have existed through stimulating the economy during outreach and training events. The awards CITiZAN earned were also unintended outcomes. CITiZAN earned the 2018 British Archaeological Award for Best Community Engagement Archaeology Project and the Charity Awards 2018 for Arts, Culture, and Heritage.

#### Longevity

The longevity of the first round of CITiZAN is challenging to assess. Earning a second round of funding indicates general success and ensures the continuation of the project. The second round of funding, CITiZAN 2019+, will continue engagement, research, and the main principles of the project. Without CITiZAN 2019+, continued engagement and research is hard to hypothesize as it depends on the volunteers’ confidence in and desires to continue recording sites on their own. CITiZAN evaluations indicate 46–50% of volunteers reported feeling confident in utilizing their site recording skills on their own to identify, monitor, and record heritage features and coastal change (Gill 2018: 12). Eighty percent of volunteers indicated they would be confident or able with assistance to conduct the same activities (Gill 2018: 12). This indicates some volunteers would be confident and able to continue engaging with coastal heritage and record and monitor sites without further assistance from CITiZAN staff. However, it does not give an indication if the volunteers would continue this work. With or without the second round of funding, the research already gathered would be publicly accessible through the CITiZAN App, interactive website, Archaeological Data Service entries, and written reports.

##### Community

**Fig. 2:**
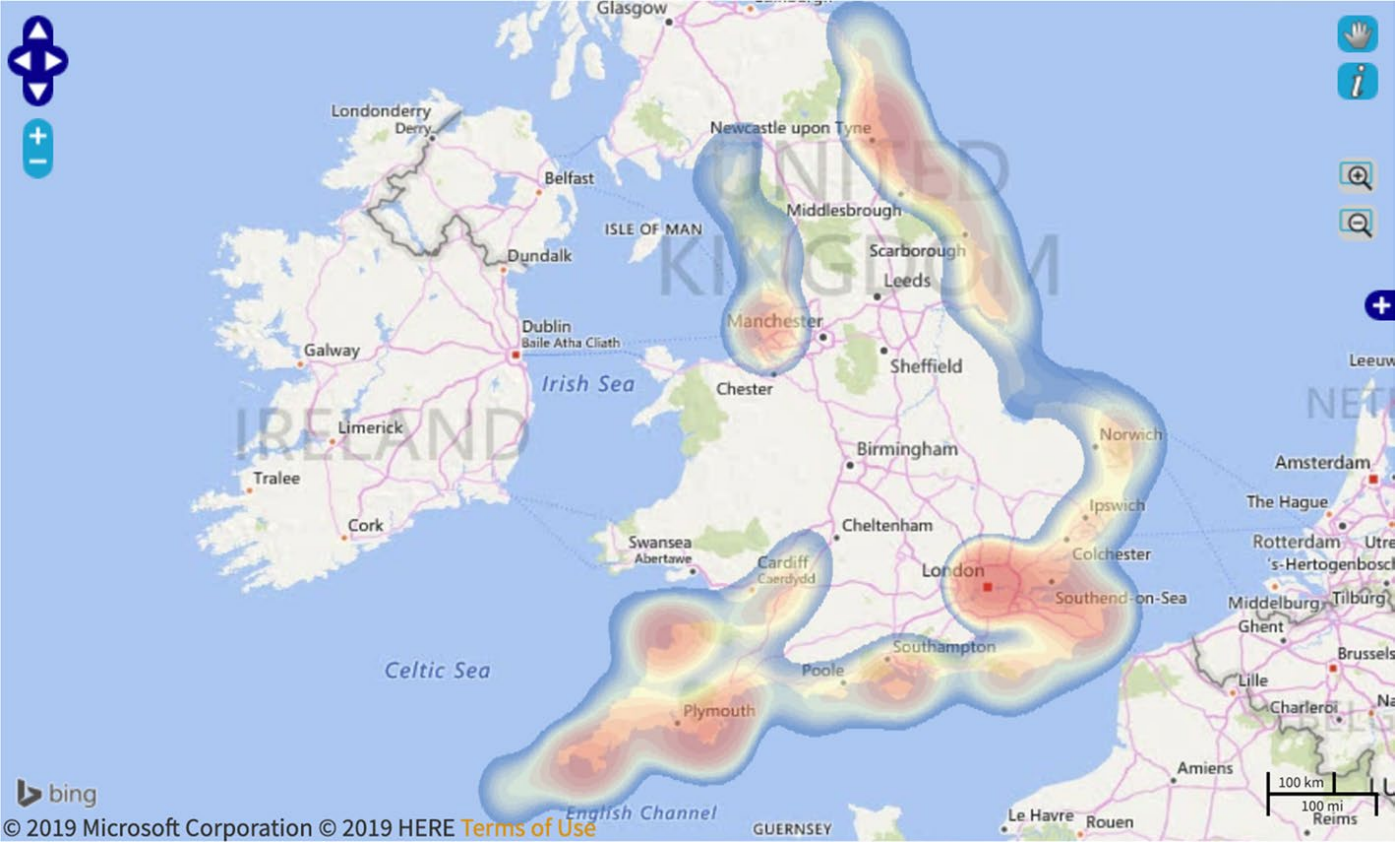
Image of the produced interactive map (CITiZAN 2019)

The social contributions of the project occurred during the period of volunteering. Unless volunteers arranged to meet outside of training or outreach sessions, these contributions ended when they stopped volunteering. The stated economic contributions likely finished at the end of the project. Economic contributions of the project may continue if people use the App or *interactive map* to locate sites of personal interest and visit them (Fig. 2).

Educationally, the events taught community members new skills and shared information about heritage in their areas. Events or training sessions generally lasted one to two days and participants could spend as much or as little time as they wanted volunteering. While this worked for many volunteers, those with limited time, access to childcare, and other constraints may not have been able to participate. This may affect the longevity of the program. Volunteers often stated in evaluations they wanted more training and practice to enhance their skills and confidence (Gill 2018). These desires decrease chances of a community member monitoring or recording sites on their own, one of the main principles of the project. Additionally, it is unclear how long the educational contributions will continue as evaluations were conducted immediately after the project.

##### Academic

The methodology CITiZAN produced, including timber vessel and non-timber vessel recording sheets, single-context sheets, and an online recording process, could be used in the future. Other programs may use the created materials and method as a starting point in their own projects. The sites added to heritage records and the produced grey literature reports are publicly available, allowing any interested party to engage with them now or into the future.

##### Heritage

The resources produced and deposited into the Archaeological Data Service (ADS) allows each Historic Environment Record (HER) to update their records accordingly. This both helps document England’s heritage and highlight important individual sites, potentially helping improve their management. Relying on the HER alone to hold the gained information poses a potential issue as it only documents sites up to the mean low tide. Some of the sites CITiZAN documented are further out to sea than this. These sites will not be included in the HER record.

## Synthesis, Assessment, and Additional Applications

Testing the framework through the use of case studies generated a number of observations about this approach to evaluating community archaeology projects. It also indicated several areas for improvement both with respect to the effectiveness of the projects but also to the framework in providing a mechanism to evaluate community, specifically maritime archaeology projects. Four key observations were made as a result of this research in terms of the effectiveness of the evaluation framework, however they all have application across the board, none specifically reflect the context of maritime community archaeological projects:

### Conclusions from the Framework

1. The ‘influencing factors’ help describe and classify the variety of community archaeology projects conducted. As previously noted, several kinds of community archaeology exist. The labels used to categorize these methods and the definitions of each label, vary between researchers and geographical locations, complicating any attempt to create definitive definitions. Rather than simply relying on these labels, this framework uses the influencing factors table to clearly categorise the projects and mitigate potential discrepancies. The influencing factors table also makes comparing a project’s basic information to another easier, as the core elements are clearly laid out.
2. The key project information presented in the influencing factors table directly relates to the project’s potential contributions*.* What these key elements represent can both limit and facilitate the contributions a project can make. For example, the duration of engagement directly impacts the relationships between involved parties. Longer durations of engagement allow for deeper bonds between community participants and project leaders, increase the opportunity for knowledge sharing, and in turn create a longer lasting impact.
3. The influencing factors table also offers a mechanism to encourage project leaders to think through the various key project elements and help construct a project with desirable impacts in mind from the outset. Project leaders or stakeholders who know the contributions they would like to make, can use the framework to identify a combination of influencing factors that will result in the desired outcomes. Potentially projects that do not have community archaeological objectives could formulate additional goals along these lines having learnt from the evaluation of successful community based maritime projects.
4. Retrospectively comparing the intended and actual project contributions, may inherently produce a positive meta-analysis. In all the case studies presented, the intended contributions were less substantial and less impactful than the actual contributions. In some cases, the intended and actual contributions only marginally varied. However, this still produces a positive meta-analysis. There are four potential reasons for this. Firstly, researchers cannot guess all project outcomes at the start of their work as the nature of research often yields unexpected results. Secondly, project leaders may not have stated or necessarily even realised, their intended outcomes fully from the outset. Thirdly, researchers may have limited the stated project ambitions even though they anticipated greater impact, in order to ensure the project’s success. Finally, none of the analysed case studies articulated negative contributions and only reported positive contributions. In addition to producing a positive meta-analysis, this prevented the demonstration of how the framework could report negative contributions. Any of these reasons will produce a positive meta-analysis when evaluating a project against its own goals.

### Conclusions from the Analysis

Analysing the three case studies revealed conclusions about community-based maritime archaeological projects:

1. In the projects reviewed here, as well as the case studies reviewed in the previous research, evaluations were rare. The reasons for this are unclear. Researchers may lack the time to reflect on their work, the tools to conduct evaluations, or value the knowledge acquired over the ability to improve the project. Alternatively, they simply may not have thought about evaluations in advance of the project as this is a relatively new practice. However, the lack of evaluations is slowly improving. For example, the recent PRAXIS-UNESCO conference ‘Heritage and our Sustainable Future’ featured an entire session on evaluating the impact of cultural heritage and presented several evaluation frameworks.
2. Information and understanding that flows freely between all involved parties, rather than just one-way from the project lead, influences the project’s outcomes. Some projects have potential for multiple dialogue, information, and comprehension between all involved. This exchange can alter the course of the project and may further impact how researchers think about or conduct their work in the future. Additional information and further reflection is needed to provide evidence for this claim.
3. Articles and reports readily detail the tangible results; however intangible heritage appears less often valued or reported on, even in situations where intangible heritage is engaged. Although intangible heritage can be more challenging, complicated, and less comfortable to work with, it holds important information and should be valued and reported.
4. The level of community engagement directly relates to the depth and longevity of contributions to the community*.* For example, ‘War in the Pacific’ operated on the partnership/co-creation level of engagement and largely contributed to the community economically, educationally, and culturally. Contributions helped reconnect communities with archaeological sites, decolonize history, and honour the lived experience of participants. These outcomes have potential for long-lasting effects. Conversely, CITiZAN functioned on the utilization level, using the community to gather information about potential sites. Community benefits largely rested in educational outcomes and archaeological site documentation, and ultimately the individuals within the community. Unless the community continues engaging with the produced materials and with CITiZAN 2019+, contributions finish at the end of the project.
5. Publicly available research facilitates longer-lasting contributions to involved stakeholders*.* All of the selected case studies in this paper and in the larger body of research, have publicly accessible information including journal articles, websites, blogs, reports, or social media pages. These sources informed the analysis as well as contributed to the longevity of the community, academic, and heritage outcomes. Conversely, dozens of projects around the world conduct community archaeology in maritime contexts outside of those presented here. Many of these only partially represent the project, lack a clear outline of the methodologies applied and contributions made, and omit other critical details, making analysis challenging and limiting their inclusion in this research. More complete publications and greater access to information would improve the assessment and quantity of case studies discussed.

### Areas for Improvement in Community Maritime Archaeology

The conclusions above indicate a number of areas where community archaeology needs improvement. Archaeologists need to consider the following:

1. The project elements listed in the influencing factors table directly impact the potential contributions a project can make. Being very mindful of the impact the influencing factors have on the project should be a key factor in project design. Selecting these factors based on the project’s intended contributions and needs will produce a more successful, thoughtful, impactful project.
2. As discussed in “Why do we need to evaluate?” section, community engagement both increases archaeology’s potential to stimulate change and can cause harm. Reflection and assessment are important elements of the critical thinking process that are frequently omitted. Rigorously analysing community archaeology provides a deeper understanding of the project. Archaeologists need to evaluate their work to understand the contributions of their project, whether they achieve their intended outcomes, and the longevity of these contributions.
3. Archaeologists need to equally report on both positive and negative contributions to truthfully depict and discuss their project. Too frequently archaeologists only report on positive contributions of their project, without pausing to consider if their project negatively impacted others. This practice undermines the growth of the field.
4. Some projects are designed to have contributions that endure well beyond the project’s completion, other projects’ affects are designed for short-term impact. Regardless of the intended contributions’ duration, archaeologists need to think about and understand how long their actual, positive and negative, contributions may last and the long-term impact on the community. Striving to comprehend the longevity of these outcomes examines the project’s consequence now and into the future. In addition, project leaders must manage community members expectations for intended contributions to avoid misleading claims or disappointment if goals are not delivered.
5. Reporting on all kinds of heritage helps depict a more complete account of the past and present than documenting only tangible or intangible. Archaeologists need to consider, value and report on tangible and intangible heritage alike.
6. Co-created projects, incorporating community members into the archaeological project from the outset and throughout, often produce more thoughtful and impactful research with longer lasting effects for all stakeholders, producing more relevant, influential, and mindful results.
7. Publications should clearly state the methodology, intended and actual contributions, and a discussion of the contributions’ longevity. Thoroughly publishing for peers, the public, and the community, provides an opportunity for learning and feedback, fostering a collaborative research environment rich with conversations and debates between all stakeholders in heritage. In turn, this stimulates theoretical, methodological, and practical advances in the discipline.

### Assessment of the Framework

As outlined in “What does an evaluation need?” section, scholars readily agree evaluations should:

1. Identify from the outset of the project, for whom the project is being conducted and why,
2. Include all stakeholders’ voices from the outset of the project, where possible,
3. Clearly identify the level and duration of community engagement,
4. Report on successes and failures,
5. Seek to understand the methodology behind achieving each outcome,
6. And offer an unbiased review.

Out of the above list, the framework presented identifies for whom the project is being conducted and why, the level and duration of engagement, and the methodology behind achieving each outcome, as well as provides an unbiased review of the case studies. The framework does have the capacity to incorporate positive and negative contributions. However, the selected case studies either did not negatively contribute or only reported positive outcomes. Therefore, the ability of the framework to report negative contributions was not demonstrated. In addition, the framework successfully navigates around the diverse labels and definitions of different types of community archaeology. The flexibility of the framework encourages its adaptation to better suit individual projects, allowing it to analyse any community archaeology project in maritime landscapes, and beyond.

The framework at present, does not incorporate the opinions of all stakeholders. The analysis presented in “case studies” section relies on publicly available information often in the form of journal articles or websites and a brief conversation with the project director. If the written sources omit voices and opinions of non-author stakeholders, then the evaluation presented in “case studies” section potentially silences these people and may unintentionally insert bias. Some projects include community members as authors, potentially mitigating this issue. Incorporating personal communications with all stakeholders into each area of the framework (the influencing factors, intended and actual contributions, and longevity) would likely prove a more successful mitigation tactic and advance the findings of this methodology. Alternatively, having project leaders, stakeholders, and funders address the framework in advance of and on completion of the projects, would no doubt produce different and potentially more significant results. However, they must be cognitive of potential biases as it may impact the validity of the evaluation. Additionally, having each beneficiary fill out the framework independently and by comparing them to each other, might reveal interesting differences in opinion, different perspectives, and provide additional avenues to explore.

Another important aspect lacking in this framework is the incorporation of gatekeepers. Gatekeeping is the action of controlling access to something (Hølleland and Niklasson 2020: 144). Gatekeepers have the power to permit or deny access. Within community archaeology, gatekeepers often are a key community member, such as an elder or leader, who has the power and access to appropriate networks to permit or deny researchers from gaining interviews or accessing information. A community gatekeeper who approves of a researcher, might give them greater permissions to access community held knowledge or other community members, than the researchers alone would achieve. This may broaden the researcher’s understanding of the community and knowledge gained. However, even when gatekeepers approve of researchers, they may intentionally only show researchers a small portion of the community, omitting portions they do not wish to show. Unintentionally the research thereby may not capture the breadth of the community (Supernant and Warrick 2014: 583). Adding a section of this framework to indicate if the project used community gatekeepers and who they are, might help illuminate the depth, perspective, and community approval of the study.

The longevity section needs further development. This section as it stands does not offer a clear evaluation of a project’s contributions. However, it does bring awareness to the issue of understanding the potential long-lasting effects of a community archaeology project. Adding more questions or discussing the project personally with involved stakeholders would likely advance the success of this aspect of the framework. Personal discussions with involved stakeholders six months or longer after the end of a project would provide valuable insight into the longevity of the contributions.

### Additional Applications

The adaptable nature of this framework allows it to be easily applied to other parts of the research process and additional subjects. Project leaders or stakeholders could use the framework when planning a project. The intended beneficiaries and contributions could be addressed from the outset. By determining which influencing factors the project needs to achieve its goals and ensuring the longevity of the contributions, the project stands more chance of success. After the project’s completion, researchers or stakeholders could fill out the actual contributions, reflecting on whether the project achieved its goals. In turn, they could assess the longevity of the contributions. This process may improve the successfulness of projects through defining their intended contributions, understanding how the influencing factors affect their project, and reflect on the actual contributions and its longevity. In addition, as discussed in “Evaluation” section, no real divide exists between land and sea. The theories, methods, and application of community archaeology transcends between maritime and terrestrial spaces. Therefore, the existing framework could be altered where needed and applied to a variety of archaeological landscapes.

This framework could also be modified to assess community engagement in heritage management. In many countries, heritage management has started incorporating communities into management strategies at the local, national, and international level (Carter 2011: 16). The resulting benefits parallel those of community in archaeology, including increasing the public value of heritage, inspiring stewards of heritage, decolonizing history, and protecting and perpetuating tangible and intangible heritage (Liston et al. 2005: 6; Chirikure and Pwiti 2008: 476; Jeffery and Parthesius 2013; Mills and Kawelu 2013: 128; Fletcher 2014: 5; Lwoga 2018: 184). Sharfman (2017) and others have advocated for heritage managers to develop ways in which the community can help guide them in developing effective, long-term management strategies for more successful heritage management and thus, the protection of the heritage itself (Sharfman 2017: 12). In a similar manner to community archaeological projects, community involvement in heritage management would benefit from evaluation to ensure that both stakeholders and the heritage are positively affected. The framework presented in “The methodology” section could be adapted for this purpose, improving understandings of the influencing factors, contributions, and longevity of involving community in heritage management.

## Conclusion

Reflection and assessment present two critical parts of research. However, archaeologists rarely pause to evaluate whether their projects achieve the intended goals, positively impact all stakeholders, and consider the longevity of their work. The presented framework provides a mechanism by which to analyse the contributions and longevity of community archaeology in maritime contexts.

The framework consists of three sections: the influencing factors, intended and actual contributions, and longevity. This process determines the kind of community archaeology conducted, compares the goals with results, and attempts to understand the longevity of these outcomes. Evaluating the framework within the context of the three case studies, demonstrates its use and how the influencing factors impact the contributions and longevity of the project.

Analysing these case studies revealed conclusions about the framework (“Conclusions from the framework” section), the nature of community archaeology projects in maritime contexts (“Conclusions from the analysis” section), as well as areas where community archaeology might improve (“Areas for improvement in community maritime archaeology” section). Archaeologists need to carefully choose influencing factors based on the desired contributions, evaluate their work, report positive and negative outcomes, and scrutinize their work beyond comparing intended and actual contributions, equally value and report tangible and intangible heritage, co-create projects alongside community members, and comprehensively publish their findings. The framework also successfully incorporates a number of necessary components of evaluations (“Assessment of the framework” section), mitigates discrepancies in labels and definitions of community archaeology, and provides a flexible methodology.

The framework provides an adaptable method for analysing community archaeology projects. This is not the only solution to the need for evaluation. This would not be feasible given that each project is unique, thus by extension every evaluation will have variations. Rather it proposes a solution and begins the dialogue, hopefully stimulating further discussion and study into this important component of research. Areas for improvement lie in incorporating the opinions of *all* stakeholders and further developing the longevity section. Archaeologists must do more to validate the value and significance of community archaeology and ensure their methods positively affect those involved. Analysing and careful consideration of the impacts of our work is paramount for the success of community archaeology, the protection of communities, and the preservation of heritage.

## Data Availability

Not applicable.
